# Tumor Necrosis Factor-A Polymorphisms and Colorectal Cancer Risk: A Meta-Analysis

**DOI:** 10.1371/journal.pone.0085187

**Published:** 2014-01-03

**Authors:** Li Min, Duo Chen, Like Qu, Chengchao Shou

**Affiliations:** 1 Key Laboratory of Carcinogenesis and Translational Research (Ministry of Education), Departments of Biochemistry and Molecular Biology, Peking University Cancer Hospital & Institute, Beijing, China; 2 Key Laboratory of Carcinogenesis and Translational Research (Ministry of Education), Departments of Epidemiology, Peking University Cancer Hospital & Institute, Beijing, China; Tor Vergata University of Rome, Italy

## Abstract

**Background and Objectives:**

Tumor necrosis factor-alpha (TNF-a) was related to inflammation and involved in the development of colorectal cancer. Polymorphisms located in *TNF-a* promoter region, such as 308G/A and 238G/A, could affect the risk of various types of cancer by regulating TNF-a production. In this study, a meta-analysis was performed to investigate the association between common polymorphisms of *TNF-a* promoter region and colorectal cancer susceptibility.

**Methods:**

Searching of several databases was performed for all publications on the association between *TNF-a* polymorphisms and colorectal cancer. Summary odds ratios (ORs) with their 95% confidence intervals (95% CIs) were calculated using random-effects models. Stratified analyses based on ethnicity and control population source were also conducted.

**Results:**

Overall, *TNF-a* 308A polymorphism showed a significant association with increased risk of colorectal cancer in worldwide populations under homozygote comparison [AA vs. GG, OR (95% CI) = 1.46 (1.07–1.97)] other than heterozygote comparison [AG vs. GG, OR (95% CI) = 1.05 (0.93–1.19)]. *TNF-a* 238A was not associated with colorectal cancer risk under homozygote or heterozygote comparisons. In stratified analysis, significant association was observed only in Western populations [AA vs. GG, OR (95% CI) = 1.39 (1.01–1.91)] other than in Eastern populations under homozygote comparison. No significant difference was observed between population-based subgroup and hospital-based subgroup.

**Conclusions:**

*TNF-a* 308A was moderately associated with an increased risk of colorectal cancer in Western populations, and *TNF-a* 238A polymorphism was not significantly associated with colorectal cancer risk.

## Introduction

Colorectal cancer is the third most commonly diagnosed cancer and the fourth leading cause of cancer-related death worldwide [1]. More than 1 million new cases of colorectal cancer were diagnosed globally every year [2], and more than 715,000 deaths were resulted from colorectal cancer in 2010, nearly twice of 490,000 in 1990 [3]. Colorectal cancer is becoming an important public health problem, especially in developed countries. In United States, both incidence rate and mortality rate of colorectal cancer rank third among all cancers in both men and women, and the lifetime risk of developing colorectal cancer is about 1 in 20 (5%) [4].

Recently, a number of risk factors for colorectal cancer were identified [5]. Among them, markers of systemic inflammation, obesity, and diabetes were found to be associated with colorectal cancer risk in prospective epidemiologic studies [6].

Biological and epidemiological studies indicated a clear association between chronic inflammation and colorectal cancer [6]–[8]. Patients with inflammatory bowel disease (IBD), including Crohn’s disease (CD) and ulcerative colitis (UC), have an increased risk of colorectal cancer [7]. Epidemiological studies also strongly suggested the involvement of genetic factors in IBD development, especially those associated with inflammation [9], [10].

Tumor necrosis factor-alpha (TNF-a) is the most important proinflammatory cytokine involved in cell growth, differentiation, and apoptosis [11], [12], which has also been reported to play a critical role in the carcinogenesis [12]. Consistent with this, a large amount of studies indicated that chronic inflammation and pro-inflammatory mediators including TNF-a may increase the risk of malignancy [9], [13]–[15]. As transcription of *TNF-a* is regulated by the promoter region of *TNF-a* gene, many studies have shown that polymorphisms located in *TNF-a* promoter region [such as 238 (rs361525), 308 (rs1800629), 857 (rs1799724), and 1031 (rs1799964)] could regulate TNF-a production, thus affecting the risk of cancers [16]–[18].

Recently, *TNF-a* 308 polymorphism was confirmed as a risk factor for a range of cancers by meta-analysis, such as gastric, breast and hepatocellular cancers [19]–[21]. However, both of the two previous meta-analyses focused on *TNF-a* 308 polymorphism claimed that it was not significantly associated with risk of colorectal cancer [22], [23], which appears in contradiction with the results of other cancers [19]–[21]. Considering the small sample size of both the two meta-analyses (1,708 and 1,742, respectively), whether *TNF-a* 308 polymorphism is associated with colorectal cancer remains inconclusive. Therefore another well-conducted, independent review of this issue was required to get a definitive conclusion.

For the association between colorectal cancer risk and other SNPs such as 238, 857, 863 and 1031, no integrated analysis has been made. In light of the heterogeneity of each published study, none of the work could achieve a reliable and consistent conclusion.

In this article, we gathered data from all previous studies of *TNF-α* polymorphisms and colorectal cancer, and a meta-analysis containing 3372 cases and 4523 controls was conducted to investigate whether *TNF-α* 308, *TNF-α* 238 promoter polymorphisms were associated with the risk of colorectal cancer.

## Materials and Methods

### Data Sources

This meta-analysis was performed according to the PRISMA guideline (Supporting information: Table S1). A comprehensive searching for all articles that had been published on the association between *TNF-a* polymorphisms and the risk of colorectal cancer was performed, using the following terms in the MEDLINE, PubMed, EMBASE: (‘Tumor Necrosis Factor-alpha’ [MeSH] OR ‘Tumor Necrosis’ OR TNF) AND (‘Polymorphism, Genetic’ [MeSH] OR polymorphism OR polymorphisms OR risk) AND (colorectal cancer). All articles were updated on Oct 25, 2013. References of all research and review articles were reviewed for additional references. Searching was conducted by two independent researchers to make sure that no published papers were missed.

### Inclusion and Exclusion Criteria

Included studies met the following criteria: (i) case–control studies on the association between *TNF-a* polymorphisms and colorectal cancer; (ii) usable genotype frequencies in cases and controls provided; and (iii) reporting outcomes and risk estimates and/or presentation of data necessary for calculating ORs with 95% CIs. We excluded studies overlapping with other studies or overlapping with data from the same authors.

### Data Extraction

Two researchers extracted the data independently. Items of the author’s last name, year of publication, country of origin, source of the study population, genotypes and numbers of cases and controls were extracted. The numbers of studies on the association between *TNF-a* 308, *TNF-a* 238 polymorphisms and the risk of colorectal cancer were 14, 4, respectively. Most of the studies used frequency-matched controls to cases by age and sex.

### Statistical Analysis

The Hardy–Weinberg equilibrium (HWE) was tested to compare the observed genotype frequencies with expected genotype frequencies in controls of all studies. ORs and 95% CIs were calculated to assess the strength of association between *TNF-a* polymorphisms and risk of colorectal cancer under homozygote comparison and heterozygote comparison. Random-effects models were used to calculate overall summary ORs and 95% CIs. Study populations were classified as Western (Europe and America) or Eastern (China, Korea, India and Iran).

The significance of the summary ORs was determined by the Z-test, in which two-sided *p*<0.05 was considered as statistically significant. The Q-statistic was calculated to examine result heterogeneity among studies, and *p*<0.1 was considered significant. The *I^2^*-statistic was also calculated to efficiently test heterogeneity [24], with *I^2^*<25%, 25–75% and >75% considered to represent low, moderate and high degree of inconsistency, respectively. Begg’s funnel plot was plotted to examine the underlying publication bias [25]. For sensitivity analysis, relatively smaller studies were excluded and the summary ORs (95% CIs) were recalculated. All analyses were done using STATA 12.0 (STATA Corporation, College Station, TX, USA).

## Results

### Characteristics of Studies

**Figure 1:**
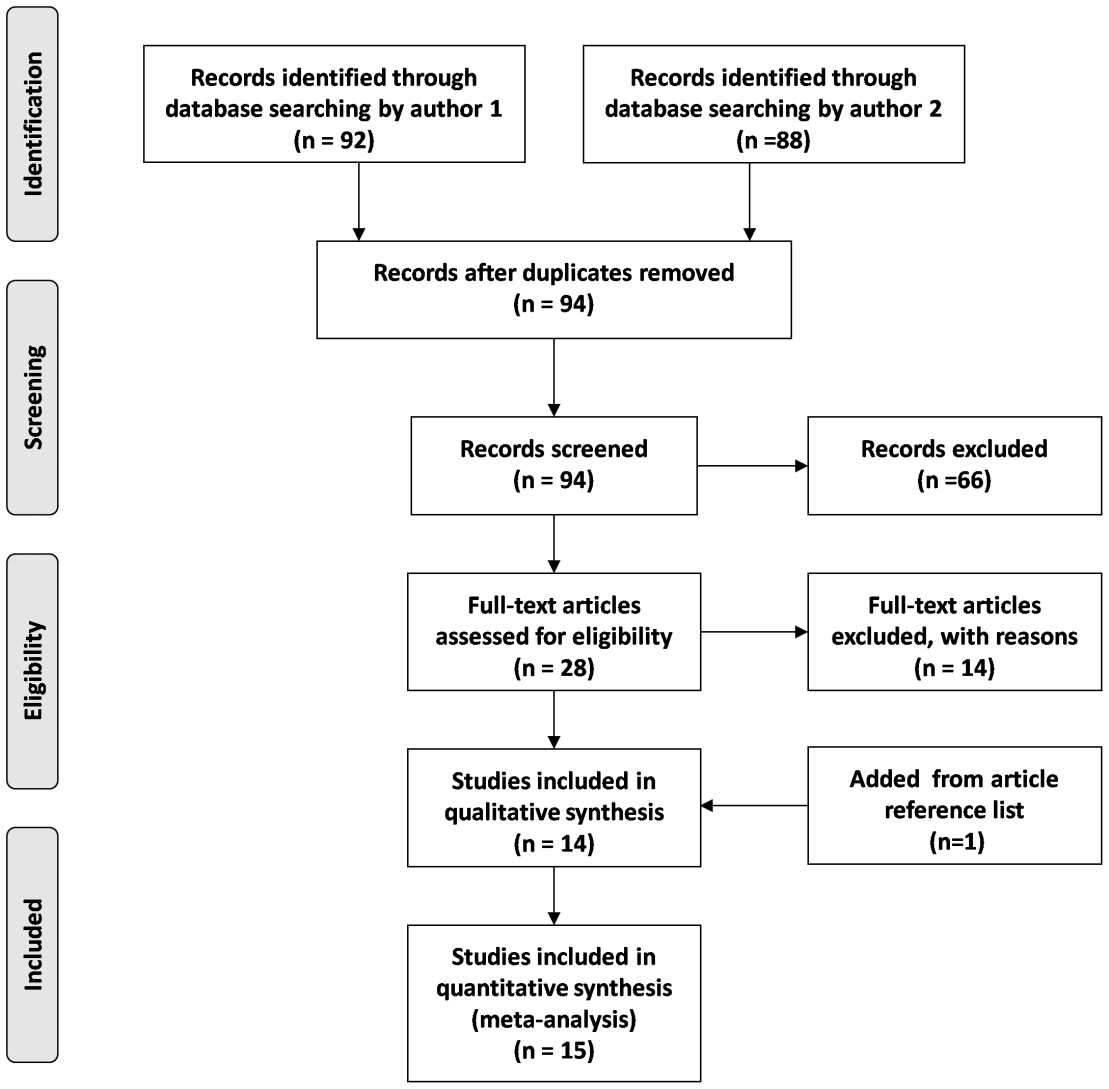
Flow diagram of included and excluded studies in meta-analysis.

In this article, 15 studies were identified to evaluate the relationship between *TNF-a* polymorphisms and risk of colorectal cancer, and a total number of 3372 cases and 4523 controls were included. Detailed screening process was shown in Figure 1. The most commonly investigated polymorphisms were *TNF-a* 308 and *TNF-a* 238, which were reported in 14 and 4 studies, respectively [2], [26]–[39]. Other SNPs, such as *TNF-a* 857, *TNF-a* 863, and *TNF-a* 1031 were also investigated in this comprehensive searching, but the corresponding sample sizes were too small to perform quantitative data synthesis. Genotype and allele distributions of *TNF-a* 308 and *TNF-a* 238 were shown in Table 1. Median frequencies of *TNF-a* 308A allele were 15.0% in Western populations and 7.1% in Eastern populations. Corresponding frequencies for *TNF-a* 238A allele were 3.5% and 7.1%, respectively.

**Table 1:** Study characteristics.

First author	year	Loc.	case	control	controlsource	308-Afrequency	*p*_HWE_	238-Afrequency	*p*_HWE_
Madani	2008	W	51	46	PB	–	–	1.09%	0.94
Park	1998	E	139	324	PB	7.07%	0.79	–	–
Jang	2001	E	27	92	HB	3.80%	0.85	7.07%	0.39
Landi	2003	W	363	320	HB	15.00%	0.70	–	–
Macarthur	2005	W	246	389	PB	23.78%	0.63	–	–
Fei	2005	E	92	132	PB	7.95%	0.78	–	–
Theodoropoulos	2006	W	222	200	PB	16.00%	0.69	–	–
Gunter	2006	W	217	202	PB	17.08%	0.68	–	–
Toth	2007	W	183	141	PB	10.64%	0.74	–	–
Wu	2008	W	156	116	PB	14.66%	0.70	3.73%	0.32
Wang	2008	E	344	669	PB	8.89%	0.77	–	–
Garrity-Park	2008	W	114	114	HB	10.53%	0.75	3.51%	0.02
Tsilidis	2009	W	204	372	PB	13.98%	0.71	–	–
Suchy	2008	W	350	350	PB	15.57%	0.69	–	–
Li	2011	E	180	180	HB	5.83%	0.60	–	–

Year: Publication year; Loc.: Location of the population; E: Eastern country; W: Western country; *p*_HWE_: *p* value of Hardy–Weinberg equilibrium, chi-square test; 308-A%: Percentage of 308-A allele frequency among controls; 238-A%: Percentage of 238-A allele frequency among controls.

### Quantitative Data Synthesis

**Figure 2:**
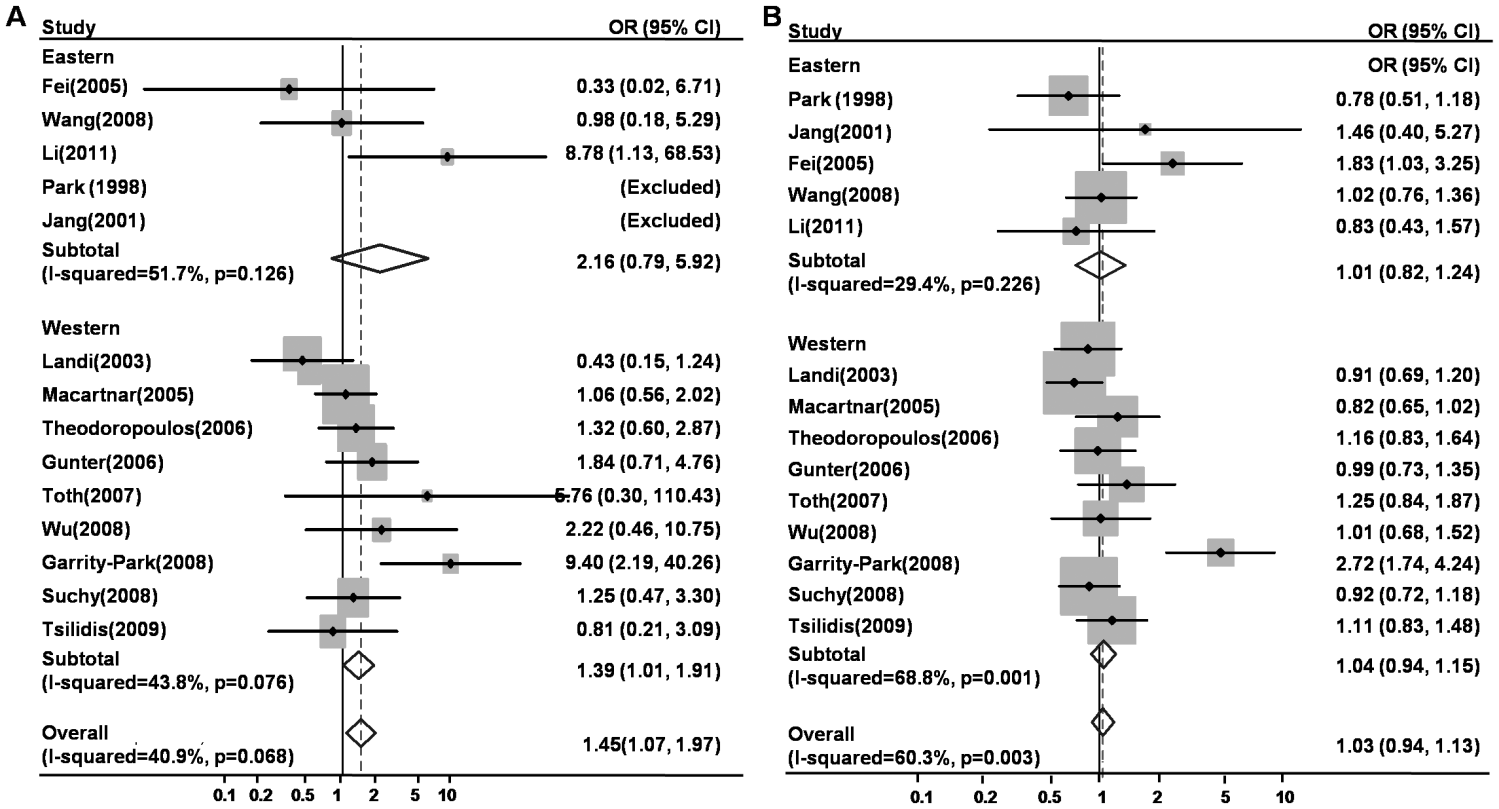
The association between *TNF*-a 308 polymorphism and risk of colorectal cancer. A: AA vs. GG, Forest plot; B: AG vs. GG, Forest plot; ORs, odds ratios.

For *TNF-a* 308G/A polymorphism, 14 studies with a total number of 2837 cases and 3601 controls were included in this analysis. Figure 2A showed the random-effects overall OR (95% CIs) of *TNF-a* 308 polymorphism under homozygote comparison [AA vs. GG, OR (95% CI) = 1.46 (1.07–1.97)]. Figure 2B showed the random-effects overall OR (95% CIs) of *TNF-a* 308 polymorphism under heterozygote comparison [AG vs. GG, OR (95% CI) = 1.05 (0.93–1.19)].

When stratified by ethnicity, for Western populations, a significant association between *TNF-a* 308 and colorectal cancer risk was observed under homozygote comparison [AA vs. GG, OR (95% CI) = 1.39 (1.01–1.91)], but not heterozygote comparison [AG vs. GG, OR (95% CI) = 1.04 (0.94–1.15)]. For Eastern populations, no significant association was found under homozygote or heterozygote comparison. Subgroups characterized by control population source (hospital-based, HB or population-based, PB), and neoplasm location (colon or rectum) were also analyzed with the same method as above (Table 2). Both homozygote and heterozygote comparisons displayed significant different risk of colorectal cancer in HB subgroup [AA vs. GG, OR (95% CI) = 2.16 (1.17–4.00)] but not in PB subgroup [AA vs. GG, OR (95% CI) = 1.28 (0.90–1.82)].

**Table 2:** Overall and group-specific summary statistics for *TNF-a* 308, *TNF-a* 238 in colorectal cancer.

SNPs	Number of studies	Comparison	Test of association[OR (95% CI)]	Test of heterogeneity		
				P-value (Z test)	*I^2^*(%)	P-value
308						
Hospital based	3	AA vs. GG	2.16 (1.17–4.00)	0.01	86%	<0.001
	4	AG vs. GG	1.19 (0.97–1.47)	0.10	84%	<0.001
Population based	9	AA vs. GG	1.28 (0.90–1.82)	0.18	0%	0.89
	9	AG vs. GG	1.00 (0.90–1.10)	0.95	23%	0.23
Colon cancer	3	AA vs. GG	1.22 (0.71–2.08)	0.48	64%	0.06
	3	AG vs. GG	0.85 (0.70–1.02)	0.09	0%	0.66
Rectal cancer	2	AA vs. GG	0.66 (0.27–1.60)	0.35	46%	0.17
	2	AG vs. GG	0.88 (0.68–1.14)	0.33	0%	0.86
238						
Hospital based	1	AA vs. GG	0.33 (0.01–8.02)	0.50	0%	0.10
	1	AG vs. GG	0.83 (0.26–2.63)	0.75	0%	<0.001
Population based	3	AA vs. GG	2.46 (0.32–19.11)	0.39	12%	0.44
	3	AG vs. GG	0.75 (0.47–1.19)	0.22	0%	0.37

CI: confidence interval; OR: odds ratio; *TNF-a*: tumor-necrosis factor-a.

**Figure 3:**
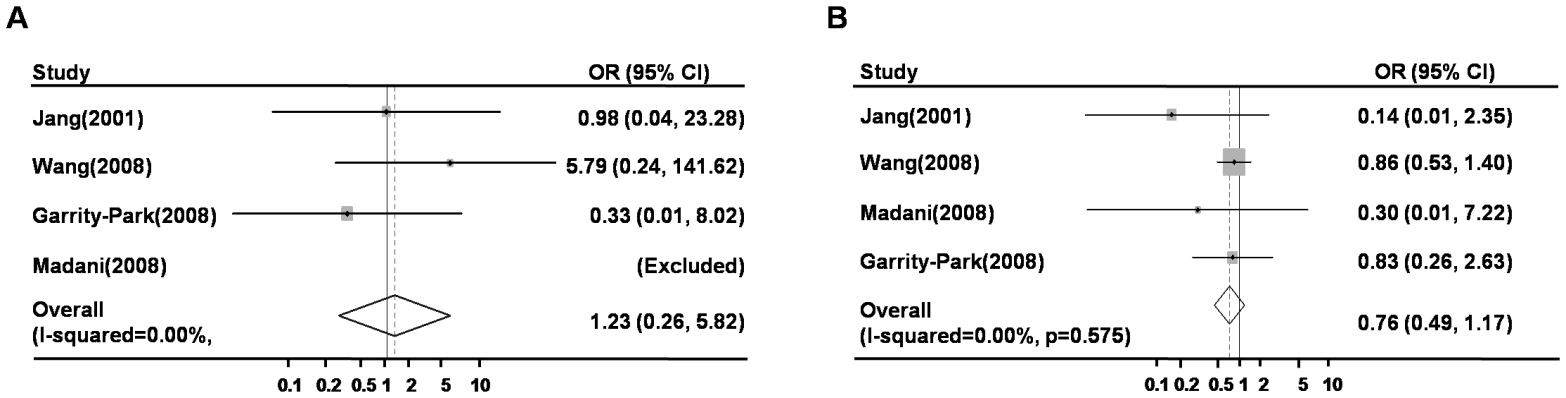
The association between *TNF*-a 238 polymorphism and risk of colorectal cancer. A: AA vs. GG, Forest plot; B: AG vs. GG, Forest plot; ORs, odds ratios.

For *TNF-a* 238 G/A polymorphism, we included 4 studies with a total number of 535 cases and 922 controls. Overall, no significant association was found between *TNF-a* 238G/A polymorphism and the risk of colorectal cancer under homozygote comparison [AA vs. GG, OR (95% CI) = 1.23 (0.24–6.23); Figure 3A] or heterozygote comparison [AG vs. GG, OR (95% CI) = 0.74 (0.47–1.17), Figure 3B]. Since there are only 4 studies recruited in this study, no stratified analysis was illustrated in the form of figures. Those results were displayed in the table along with results of *TNF-a* 308 instead (Table 2).

### Test of Heterogeneity

For the overall meta-analysis, the Q-statistic was significant and *I^2^* showed a moderate variation for *TNF-a* 308 (AA vs. GG, *I^2^* = 40.9%, *p* = 0.07; AG vs. GG, *I^2^* = 60.3%, *p* = 0.01), and no variation for *TNF-a* 238 (AA vs. GG, *I^2^* = 0.0%, *p* = 0.46; AG vs. GG, *I^2^* = 0.0%, *p* = 0.58).

In the stratified analyses of *TNF-a* 308, *I^2^* showed moderate variations in both Eastern (AA vs. GG, *I^2^* = 51.7%, *p* = 0.13) and Western populations (AA vs. GG, *I^2^* = 43.8%, *p* = 0.08). For PB and HB subgroups, *I^2^* showed no variation (AA vs. GG, *I^2^* = 0.0%, *p* = 0.884) and a strong variation (AA vs. GG, *I^2^* = 86.9%, *p*<0.001), respectively, which indicated that hospital-based studies might be important source of heterogeneity. Moreover, variation in the frequency of this *TNF-a* 308 polymorphism among the populations or some unidentified factors may also be the source of heterogeneity.

### Sensitivity Analysis and Publication Bias

To further strengthen our conclusions, the sensitivity analysis was performed. A single relatively small study involved in the meta-analysis was excluded each time. For *TNF-a* 308, the corresponding pooled ORs were not changed significantly (ORs ranged from 1.20 to 1.54 under homozygote comparison, from 1.00 to 1.12 under heterozygote comparison, detailed data not shown), indicating that our results were statistically robust. Sensitivity analysis was not performed for *TNF-a* 238 because there were only 4 studies included.

**Figure 4:**
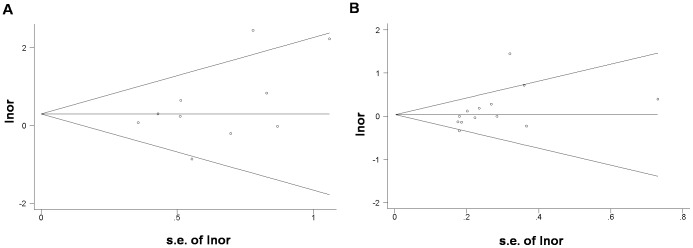
Publication bias tests for the association between *TNF*-a 308 polymorphism and colorectal cancer. A: AA vs. GG, Begg’s funnel plots; B: AG vs. GG, Begg’s funnel plots.

For publication bias investigation, Begg’s funnel plot and Egger’s weighted regression were performed for the association between *TNF-a* polymorphisms and colorectal cancer. No evidence for bias was detected in *TNF-a* 308 polymorphism under homozygote comparison (Figure 4A. Begg’s test p = 0.325, Egger’s test p = 0.180). However, evidence of publication bias was found in the heterozygote comparison (Figure 4B. Begg’s test p = 0.024, Egger’s test p = 0.063). Publication bias investigation was not performed for *TNF-a* 238 because there were only 4 studies included.

## Discussion

Inflammation is one of key factors involved in carcinogenesis, and people with inflammatory bowel disease are at high risk of colorectal cancer [7]. Therefore polymorphisms of inflammation-related genes have been regarded as potential sources of cancer risk biomarkers [5]. TNF-a, the most extensively studied inflammatory factor in cancer, was proved to affect colorectal tumorigenesis through different pathways [11], [12]. Previous studies indicated that TNF-a-related cellular functions were greatly influenced by polymorphisms in the promoter region of *TNF-a* gene [16]–[18].

308G/A is the most commonly studied SNP site of *TNF-a* promoter region. Various meta-analyses indicated that *TNF-a* 308A polymorphism was associated with an elevated risk of gastric cancer [19], breast cancer [20], cervical cancer [40] and lung cancer [41]. However, for colorectal cancer, two previous meta-analyses reported that *TNF-a* 308 polymorphism was not associated with colorectal cancer risk [22], [23], and one of these two meta-analyses also demonstrated that *TNF-a* 308A polymorphism was associated with reduced risk of colorectal cancer in Western population [22]. Additionally, another systematic review focused on field synopsis of genetic association also included a meta-analysis reporting *TNF-a* 308 polymorphism was not associated with colorectal cancer risk [42]. These results were distinct from nearly all other types of cancer, and were hardly explained by the mechanism of TNF-a. In our study, 2725 cases and 3369 controls from 14 independent studies were included, and the previous conclusion was not supported by our study. According to our study, *TNF-a* 308A polymorphism was associated with an elevated risk of colorectal cancer under homozygote comparison. This result was in accordance with the meta-analysis of other cancers, and also could be explained by the previous mechanistic study of TNF-a [43], which reported that in humans, the presence of a promoter polymorphism of *TNF-a* 308A is associated with increased plasma TNF-a concentration, which may lead to an increased risk of cancers.

However, the association between *TNF-a* 308 polymorphism and colorectal cancer risk became less significant under heterozygote comparison. Compared with AA genotype, the lack of association between *TNF-a* 308 AG genotype and increased risk of colorectal cancer could be explained by haploinsufficiency effect, that is, the elevated plasma TNF-a concentrations resulted from a single *TNF-a* 308 A allele might be not high enough to strongly affect cancer risk. Sensitivity analysis indicated that when excluded each study involved in the meta-analysis, ORs under heterozygote comparison stayed over 1.00, suggesting that if more studies were included in the future analysis, the association between *TNF-a* 308 AG genotype and increased risk of colorectal cancer might also become statistically significant.

Comparable ORs were observed when analyses were stratified to Eastern and Western populations, indicating that there was no obvious race-specific effect in this regard. However, since the sample size of Eastern populations is relatively small, the association between *TNF-a* 308A and risk of colorectal cancer was not significant. Strictly, we can only conclude that *TNF-a* 308A was moderately associated with an increased risk of colorectal cancer in Western populations.

As it is shown in Table 2, AA vs. GG comparison results were significant in HB subgroup but not in PB subgroup, which suggested that control source might affect the association between *TNF-a* 308 polymorphism and colorectal risk in Hospital-Based study. Together, we concluded that *TNF-a* 308A polymorphism was a general risk factor of colorectal cancer.

*TNF-a* 238G/A is the second commonly studied SNP site following 308 among all polymorphisms located in the promoter region of *TNF-a* gene. However, recently no integrated analysis has been made to get a definitive conclusion of whether *TNF-a* 238G/A is associated with colorectal cancer. Up to now, among all publications, 4 studies investigated the association between *TNF-a* 238 polymorphism and risk of colorectal cancer, but yielded contradictory results. In our meta-analysis, 535 cases and 922 controls were enrolled and we found that *TNF-a* 238 polymorphism was not significantly associated with colorectal cancer risk Previously, a meta-analysis including 34 studies with 34,679 cases and 41,186 controls reported that no significant association was found between the *TNF-a* 238 polymorphism and the overall cancer risk [44], which is in conformity with our present results. However, the number of current studies on *TNF-a* 238 polymorphism and risk of colorectal cancer is relatively small, thus investigations involving more cases are needed in the future.

Genome-Wide Association Study (GWAS) is also a powerful methodology in genetic studies. As for GWAS studies of colorectal cancer [45]–[48], there is no evidence of association with the *TNF-a* region. Compared with GWAS studies, meta-analyses are easy to be influenced by sample heterogeneity and inaccuracies in a single study. However, GWAS studies also have demerits. To control the cost, at the first step of GWAS studies, researchers always screen for significant SNP in the whole genome including more than 500,000 SNPs in a small population, and it couldn’t have enough power to identify all SNPs associated with a disease. For example, one GWAS study only identified six possible risk alleles for colorectal cancer [45], which seems too few for such a complex disease. So no evidence of association with the *TNF-a* region derived from GWAS does not necessarily mean that all SNPs in this region have no association with risk of colorectal cancer. We speculated that, similar to Barton et al. reported on Rheumatoid Arthritis [49], expanding the screen criteria at the first step of GWAS may improve the testing power and generate more SNPs associated with colorectal cancer, and *TNF-a* 308 polymorphism might be among them. However, for the original GWAS data are not published, we couldn’t verify such speculation. On the other hand, a well-conducted meta-analysis is equal to a multi-centered, randomized, and controlled study, which could also generate a credible conclusion. Even in the GWAS era, combining available information to generate an integrated result is still reasonable and can save a considerable amount of resources.

Several limitations of this study should also be addressed. Firstly, the sample size was relatively small for stratified analyses, which weakened our conclusions, especially under homozygote comparison. Therefore more studies were needed to be included to obtain a more reliable result. Secondly, detailed information of individual level was lacking in this analysis, for which many stratified analyses were not able to be performed. If individual raw data were available, effect induced by age, gender, drug use and other environmental factors could also be investigated. Thirdly, cytokines, such as interleukins, transform growth factor, tumor necrosis factor, may exert interacting functions with each other, so SNPs of other cytokines should also be taken into account to conclude a true effect if possible. Fourthly, results of GWAS studies were not included because the raw data had not been published. Additionally, gene-gene, gene-phenotype, and gene-environment interactions should be considered.

In summary, this meta-analysis had pooled all the available data related to *TNF-a* polymorphisms and colorectal cancer, and indicated that *TNF-a 308* was moderately associated with an increased risk of colorectal cancer in Western populations. *TNF-a* 238 polymorphism was not significantly associated with colorectal cancer risk. Additionally, large well-designed cohort studies would warrant to confirm this conclusion, and to fully understand the molecular mechanism of colorectal cancer. Further prospective studies in combination with analyses of other cytokines and environmental factors are also required.

## Supporting Information

Table S1 **PRISMA Checklist of items to include when reporting a systematic review or meta-analysis.**
(DOC)
